# The double-edged sword effect of conscientiousness on the work engagement of medical staff in Chinese public hospitals

**DOI:** 10.3389/fpubh.2025.1506454

**Published:** 2025-01-30

**Authors:** Hui Sun, Xuelu Hua, Shibing Wu, Ling Sun

**Affiliations:** ^1^School of Business, Jiangsu Ocean University, Lianyungang, China; ^2^Department of Burns and Plastic Surgery, Lianyungang Hospital of Traditional Chinese Medicine, Lianyungang, China; ^3^School of Environmental Engineering, Xuzhou Institute of Technology, Xuzhou, China

**Keywords:** conscientiousness, work engagement, thriving at work, workload, perceived insider status, medical staff

## Abstract

**Background:**

In China, medical staff in public hospitals are the primary providers of healthcare services. Their work engagement directly affects patients’ perceptions of the level and quality of their medical services. Conscientiousness has a significant impact on the work engagement of medical staff, but the mechanism between the two is not yet clear.

**Objective:**

This study examined the double-edged sword effect of conscientiousness on medical staff work engagement, with a particular focus on exploring the mediating effects of thriving at work (TAW) and workload, as well as the moderating effect of perceived insider status (PIS).

**Methods:**

The study sample was drawn from four public hospitals in the Jiangsu Province, Shanghai City, and Zhejiang Province. Data were statistically analyzed using SPSS, and mediation and moderations effects tested were conducted through the Bootstrap method.

**Results:**

The results demonstrate the following: (1) conscientiousness has a positive effect on the work engagement of medical staff; (2) conscientiousness has a positive effect on work engagement through thriving at work (TAW), and a negative effect on work engagement through workloads; (3) perceived insider status (PIS) moderates the relationship between conscientiousness and TAW, and the relationship between conscientiousness and workloads; and (4) PIS moderates the indirect effect of conscientiousness on work engagement through TAW, whereas the mediated relationship is strengthened when medical staff members exhibit a higher PIS. Simultaneously, PIS moderates the indirect effect of conscientiousness on work engagement through workload, and the mediating relationship is weakened for medical staff members who have a higher PIS.

**Conclusion:**

This study provides a balanced and dialectical understanding of the impact of conscientiousness, providing significant reference for enhancing the work engagement of medical staff in public hospitals.

## 1 Introduction

In China, medical staff in public hospitals are the healthcare service providers for the vast majority of the population. Relevant data indicate that medical staff in tertiary hospitals in China work an average of 51.05 h per week, whereas medical staff in secondary hospitals work an average of 51.13 h per week (1), less than one-quarter of medical staff are able to take their statutory annual leave. In addition to long working hours, factors such as poor communication between doctors and patients, and differences in patients’ understanding of health have led to increasingly tense doctor-patient relationships in China. The number of medical disputes has been increasing annually, and medical problems occur occasionally (2). Therefore, in this context, medical staff need heightened conscientiousness to overcome external environmental pressures and internal mental stressors.

Previous researches have mainly shown the positive impacts of conscientiousness in the workplace such as improving job performance (3), enhancing subjective well-being (4), bringing job satisfaction (5), and proactive organizational behavior (6). But some scholars have also pointed out the potential negative impact of conscientiousness. For example, Liu et al. (7) noted that employees with higher conscientiousness tend to spend a considerable amount of time, investing high effort into their work to achieve high performance, which can make them more susceptible to feelings of stress, tension, and depression. These studies have enriched the understanding of the impact of conscientiousness from the perspectives of work behaviors and outcomes, but they have not paid attention to individuals’ work engagement. Work engagement reflects employee’s work states and serves as a crucial factor for organizations to achieve performance breakthroughs (8). The level of work engagement among medical staff in public hospitals directly affects patient perceptions of the level and quality of medical services, playing a positive role in improving clinical treatment outcomes and enhancing patient satisfaction with medical care (9). In this context, investigating whether conscientiousness among medical staff in public hospitals influences their work engagement and understanding how this influence occurs is not only a realistic problem, but also a theoretical inquiry into the personality traits of medical staff in the specific context of China’s national conditions. Considering that work engagement is the result of the combined action of multiple factors, and based on the above-mentioned contradictory research conclusions, we boldly assume that conscientiousness is likely a double-edged sword, with its intensity having a dual effect on an individual’s work engagement. To clarify the dual nature of conscientiousness and the internal mechanisms through which it influences work engagement of medical staff, this study introduced the conservation of resources (COR) theory.

COR theory is based on the primary assumption that individuals have an inherent tendency to acquire, build, or preserve high-quality resources (10). Based on this assumption, individuals typically experience two effect mechanisms in resource processing: resource gain and resource loss spirals. Conscientiousness has two potential outcomes regarding its impact on medical staff work engagement. On the one hand, high conscientiousness provides internal resources that awaken medical staff’s achievement needs, facilitating the utilization of personal traits to ignite work vitality and sustained learning, which leads to a state of TAW and triggers a spiral of resources. This in turn prompts greater effort and a heightened display of work engagement in daily tasks. On the other hand, owing to their intense focus on work tasks and high self-demand, medical staff with high conscientiousness are likely to experience extended working hours. This inevitably contributes to a heightened workload, initiating a resource loss spiral, and consequently reducing the willingness to engage in work.

Furthermore, the social exchange theory posits a relationship between reciprocal benefits between employees and organizations. When employees perceive a strong and mutually recognized connection with an organization, they identify as “insiders.” PIS has a direct and profound impact on employee work autonomy and workplace behavior (11). Therefore, to explore both value-adding and value-depleting mechanisms of conscientiousness in work engagement, this study introduced PIS as a boundary condition to examine whether it plays a moderating role in these mechanisms.

In summary, based on COR and social exchange theory, this study investigated the mechanism of conscientiousness on the work engagement of medical staff. The potential contributions of this study are threefold. First, we analyzes the benefits and drawbacks of conscientiousness. It provides empirical support that contradicts the consensus regarding the generally favorable effects of such personality. Second, we apply the COR framework to identify TAW and workload as distinct internal mechanisms in order to clarify how conscientiousness affects work engagement of medical staff. By simultaneously capturing the resource gain and loss processes that originate from conscientiousness, our research goes beyond most previous literature, which only considers the impact of conscientiousness on individuals’ work outcomes and work status through a single resource gain path (7). This contributes to a more nuanced understanding of the impact of conscientiousness in the theoretical realm, supplementing the existing research on this trait. Third, by highlighting PIS as an important boundary condition, this study identifies which kinds of medical staff can amplify the positive influence of conscientiousness and mitigate its negative impact. Previous research has found that the effectiveness of conscientiousness highly depends on contextual factors (3). However, it has largely overlooks the possibility of these impacts being contingent upon medical staffs’ individual differences.

## 2 Theory and hypothesis

### 2.1 The gain path between conscientiousness and work engagement: the role of TAW

Conscientiousness is related to purposefulness, striving to achieve goals, high self-discipline, and planning- and task-orientation (12). Work engagement is a positive behavioral state in which employees integrate physical, cognitive, and emotional aspects into their work (13). TAW was proposed by Spreitzer et al. (14) and it refers to the simultaneous experience of “vitality” and “learning” by employees in their work. It is both a psychological state and a subjective perception of work.

The COR theory has been one of the most influential theories in organizational behavior research over the past 30 years, exploring the employees’ psychological and behavioral motivations (15). It is commonly applied to research topics, such as self-regulation in the workplace (16), interpersonal interaction (17), and work–family conflict (18), providing theoretical explanations for a wide range of organizational psychological and behavioral phenomena.

COR theory suggests that individuals strive to preserve and maintain existing resources while continuously acquiring new resources (15). Conscientious employees place greater emphasis on personal achievement, exert more effort at work, set higher goals, and strive to achieve them (19). Furthermore, traditional Chinese culture emphasizes a sense of responsibility and spirit of dedication, especially in the medical field, where medical staff often see their work as a mission. This value system prompts them to transform their intrinsic sense of responsibility and meaning perception into more emotional resources, exhibit more positive and proactive behaviors (4), and contribute to the improvement of organizational performance. Sethi & Kaur (20) suggested that conscientiousness was related to increased work engagement.

According to COR theory, driven by intrinsic motivation and achievement needs, conscientiousness prompts employees to actively and purposefully focus on their work tasks. This task focus enables TAW directly because purposeful participation in work tasks facilitates the learning of new things and injects employees with positive energy (14). Consistent with this reasoning, Alikaj et al. (21) discovered that proactive personality traits, such as conscientiousness, can promote TAW.

Furthermore, according to COR theory, TAW encourage employees to continuously accumulate high-quality resources, motivating them to invest more effort in the work they identify with (22). When individuals have abundant resources, they can prevent resource depletion more effectively, actively acquire new resources, and consequently achieve a more positive work experience. According to this logic, employees who reach the TAW state can gain a sense of competence by completing challenging tasks, which increases their work resources and induces a positive cycle. Consistent with this reasoning, Imran et al. (23) empirically found that TAW positively impacts work engagement.

Conscientiousness endows employees with a stronger motivation to learn, and it actively reinforces their work motivation (24), providing more intrinsic meaning to their work. In turn, this promotes self-thriving at work through perceiving vitality and embracing learning changes in the job. When employees enter a state of vibrant and energized TAW, they may seize various opportunities for learning, growth, and development, which compels them to become more engaged in their work. Therefore, we propose the following hypothesis:

*H*1: Conscientiousness has a positive indirect effect on work engagement through TAW.

### 2.2 The loss path between conscientiousness and work engagement: the role of workload

Workload refers to the number and variety of tasks that employees are required to perform (25). It also represents the physiological and psychological costs that employees invest in accomplishing their tasks (26).

Conscientious employees are more inclined to allocate personal resources to meet job performance requirements, and they are willing to invest energy in overcoming work-related challenges (27). According to management practices in China, medical staff often have a higher sense of conscientiousness compared to other professionals (1). When balancing work, rest, and family time, they tend to prioritize work. In addition, considering factors such as patient needs and teamwork, they may voluntarily accept additional tasks, be willing to work overtime or adjust work arrangements when needed, as well as be willing to take on more responsibilities and contribute more to their work, thus presenting a workload state.

The COR theory suggests that the total amount of resources possessed by an individual is limited. When facing a high workload, an imbalance among an individual’s limited resources can result in emotional resource depletion across multiple roles, conflicting with their pursuit of work–family harmony, which exacerbates their role stress (28) and triggers a resource loss spiral effect. To change the conflict caused by incompatible roles, employees may reallocate resources and choose conventional ways of handling work, thereby weakening their willingness to engage in the work. Most studies on workload have indicated that an excessive workload negatively affects employees’ emotions and outcomes. Wang et al. (29) discovered that an elevated workload negatively affects work engagement. Therefore, we propose the following hypothesis:

*H*2: Conscientiousness has a negative indirect effect on work engagement through workload.

### 2.3 Moderating effect of PIS

In the field of organizational behavior, many scholars have started using the concepts of “insiders” and “outsiders” based on social exchange theory to explore the relationship between employees and organizations. Social exchange theory is a sociological theory proposed by Blau (30) to study the behavioral relationships between individuals. This is one of the most influential conceptual paradigms for understanding workplace behavior in organizational behavior (31). PIS refers to an individual’s subjective perception of being acknowledged as a member of an organization, reflecting a sense of belonging and influencing individual cognition, emotions, and behavioral tendencies toward the organization (11). In the context of Chinese culture, employees attach great importance to being accepted by their organization. Becoming an “insider” is of great significance to employees, and it directly affects their performance (31).

According to Tracy and Judith (32), when an individual has high PIS, it is easier to stimulate individual learning behaviors. According to the social exchange theory, when employees perceive recognition from an organization, a strong sense of ownership is generated. They believe that they need to make additional efforts to attract more work resources to the organization, which enhances their vitality and learning status. However, individuals with low PIS position themselves outside the organization. They perceive work as a task to be completed and do not recognize the importance of actively acquiring various knowledge and skills or being actively involved in change, thereby reducing the TAW likelihood. Therefore, we propose the following hypothesis:

*H*3: PIS positively moderates the relationship between conscientiousness and TAW; the positive relationship between conscientiousness and TAW is stronger among employees with higher PIS than among those with lower one.

According to social exchange theory, social exchange is reciprocal behavior. Employees’ PIS is based on the foundation of high-quality relationships with the organization and its members (33). Employees with higher PIS perceive the organization as another “family,” which leads to experiencing psychological job security (34); this provides them with confidence that they can receive help from others when encountering problems at work (35). Therefore, when employees have a higher PIS, they are more likely to seek help from the organization and collaborate with colleagues to share and solve the workload together, especially in the face of a high workload (36), thereby decomposing the workload to a certain extent and weakening the positive effect of conscientiousness on individual workload. In contrast, when employees have lower PIS, it means that they often “float” outside the organization (11). In such cases, high workload can lead to negative physical and mental experiences. This situation is worse than simply being driven by conscientiousness to handle high workloads. In this scenario, the positive impact of conscientiousness on individual workload was expected to increase. Therefore, we propose the following hypothesis:

*H*4: PIS negatively moderates the relationship between conscientiousness and workload. The positive relationship between conscientiousness and workload is weaker among employees with higher PIS than among those with lower one.

Building on Hypotheses 1 and 3, this study suggests that PIS positively moderates the mediating effect of TAW on the relationship between conscientiousness and work engagement. Employees with higher PIS are granted greater autonomy in their inherent roles. Conscientiousness drives them to actively acquire the cognitive, knowledge, and skill-based resources needed for work, thereby enhancing their work learning and vitality, igniting a positive work attitude and consequently leading to wholehearted work engagement. Similarly, in line with Hypotheses 2 and 4, this study suggests that PIS negatively moderates the mediating effect of workload on the relationship between conscientiousness and work engagement. When employees have a higher PIS, the self-discipline and sense of responsibility brought about by conscientiousness prompt them to actively seek organizational support when facing a high workload, aiming to quickly recover from resource loss and fully immerse themselves in their work. Thus, we propose the following hypotheses:

*H*5: PIS moderates the indirect effect of conscientiousness on work engagement through TAW so that the mediated relationship is strengthened when an employee has a higher level of PIS.

*H*6: PIS moderates the indirect effect of conscientiousness on work engagement through workload so that the mediated relationship is weakened when an employee has a higher level of PIS.

**Figure 1:**
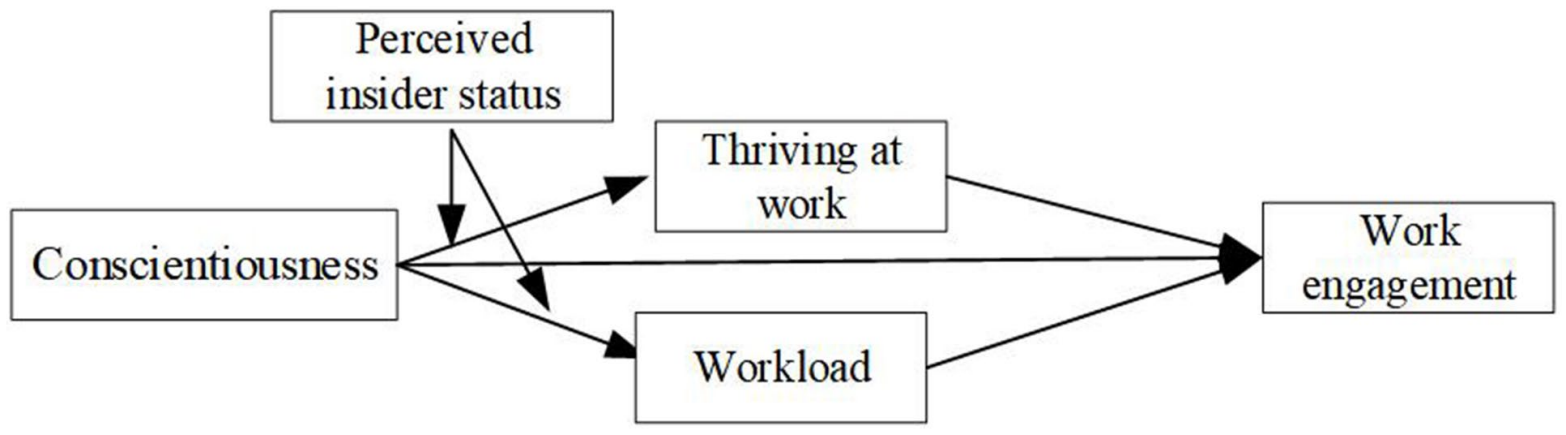
Research framework.

The conceptual model of this study is presented in Figure 1.

## 3 Materials and methods

### 3.1 Participants and procedures

The study sample was drawn from four public hospitals in the Jiangsu Province, Shanghai City, and Zhejiang Province. The research team distributed the survey questionnaires to the HRM departments. Questionnaires were randomly distributed onsite in the hospitals. Medical staff were free to decide whether to participate in the survey. We assured the respondents that the questionnaire would be anonymous and that the research data would be used solely for academic purposes, encouraging them to respond based on their genuine thoughts. To avoid common method variance (CMV), we distributed the questionnaires at separate times with a 1-month interval (37). At the initial time point, Questionnaire A was distributed, in which employees completed items on demographic variables, conscientiousness, and PIS. One month later, questionnaire B was distributed among the valid samples, which included surveys on TAW and workload. In another month, the survey questionnaire on work engagement was completed. We meticulously matched prenumbered questionnaires following a strict procedure, with the assistance of the relevant hospital HRM departments. After excluding the invalid questionnaires with responses exhibiting patterns or multiple selections, 322 valid questionnaires were paired.

Of the 322 valid questionnaires, 41.30% were completed by male participants. Doctors accounted for 56.52%, whereas nurses accounted for 43.48%. The majority of respondents were aged between 31 and 40 years (39.13%) and 41 and 50 years (30.43%). Regarding education, the majority of the participants held a bachelor’s degree (63.04%), followed by a master’s degree (26.08%). In terms of organizational tenure, the primary ranges were 5–10 years (26.08%) and 10–20 years (36.95%).

### 3.2 Measures

The questionnaire used a 5-point Likert-type scaling method as follows:

Conscientiousness: A 12-item scale from the NEO Personality Inventory developed by McCrae and Costa (38) was used to measure conscientiousness. A sample item was “I am able to get organized” (*α* = 0.819).

TAW: A 10-item scale developed by Spreitzer et al. (14) was used to measure the TAW. A sample item was “I feel alive and vital” (*α* = 0.737).

Work Engagement: A 17-item scale developed by Schaufeli et al. (39) was used to measure work engagement. A sample item was “I am immersed in my work” (*α* = 0.863).

Workload: A 5-item scale developed by Spector and Jex (25) was used to measure workload. A sample item was “I have to speed up in order to complete the work” (*α* = 0.865).

PIS: A 6-item scale developed by Stamper and Masterson (11) was used to measure PIS. A sample item was “I feel very much a part of my work organization” (α = 0.772).

Control Variables: Based on previous research, we controlled for the medical staff’s sex, age, education, position, and organizational tenure.

## 4 Data analysis and results

### 4.1 Confirmatory factor analysis and common method bias control

This study utilized Amos software for confirmatory factor analysis (CFA) to evaluate the discriminant validity of the five-factor model, including conscientiousness, TAW, workload, work engagement, and PIS. Table 1 indicates that the data fit for the five-factor model was better than that for the alternative models, suggesting its suitability for further research.

**Table 1:** Fit of confirmatory factor analysis.

Factor model	χ^2^/df	CFI	TLI	RMSEA	SRMR
Baseline model Five-factor model	2.012	0.935	0.942	0.060	0.049
Alternative models Four-factor model	3.031	0.860	0.900	0.094	0.076
Three-factor model	4.865	0.855	0.841	0.100	0.092
Two-factor model	5.234	0.807	0.818	0.125	0.143

*N = 322. Four-factor model = Conscientiousness and workload loading on one common factor; Three-factor model = Conscientiousness, TAW, and workload on one common factor; Two-factor model = Conscientiousness, TAW, workload, and work engagement loading on one common factor.*

Rigorous procedural controls were implemented before and during the survey to mitigate the effects of CMV on the study results (37). However, as the same participants were involved in different measurements, Harman’s single-factor test was used to examine the presence of CMV. The results show that 10 factors were extracted from the nonrotating principal component analysis, accounting for 73.45% of the total variance, among which the first factor explained 20.15%. This percentage does not constitute the majority, indicating that the CMV does not influence the results.

### 4.2 Descriptive statistics and correlations

As shown in Table 2, conscientiousness was significantly and positively correlated with work engagement (*r* = 0.480, *p* < 0.001), workload (*r* = 0.186, *p* < 0.05), and TAW (*r* = 0.410, *p* < 0.01). Additionally, TAW is significantly positively correlated with work engagement (*r* = 0.509, *p* < 0.01), whereas workload is significantly and negatively correlated with work engagement (*r* = −0.298, *p* < 0.05). These results provide the initial evidence to test our hypotheses.

**Table 2:** Correlation coefficient and descriptive statistical analysis of variables.

Variables	1	2	3	4	5	6	7	8	9	10
1. Gender	1									
2. Age	−0.081	1								
3. Education	−0.014	0.223	1							
4. organizational tenure	0.162	0.431^**^	0.074	1						
5. Position	0.158	0.145	0.160	0.122	1					
6. Conscientiousness	0.011	−0.100	0.042	0.181	0.065	1				
7. TAW	0.047	0.102	−0.037	0.095	−0.084	0.410^**^	1			
8. Work Engagement	0.133	0.056	0.013	0.212	0.114	0.480^***^	0.509^**^	1		
9. Workload	0.185	−0.147	−0.081	−0.053	−0.072	0.295^*^	0.182	−0.298^*^	1	
10.PIS	−0.031	0.162	0.023	−0.08	−0.251	0.186^*^	0.594^**^	0.249	0.160	1
M	1.587	2.46	2.48	2.72	1.435	3.359	3.509	3.518	2.543	3.286
SD	0.501	0.832	0.679	0.886	0.501	0.3978	0.264	0.415	0.624	0.691

*N* = 322. *p<0.05. **p<0.01. ***p<0.001.

### 4.3 Hypothesis testing

#### 4.3.1 Mediation effects test

The PROCESS macro was used to test the research models. Model 4 was selected using 5,000 bootstrap samples and a 95% confidence interval (CI). First, we tested the mediating effect of TAW. As shown in Table 3, conscientiousness had a significant positive impact on both TAW (*β* = 0.303, 95% CI [0.076, 0.471]) and work engagement (*β* = 0.591, 95% CI [0.302, 0.880]). In addition, the positive indirect effect of conscientiousness on work engagement via TAW was significant (*β* = 0.326, 95% CI [0.120, 0.731]). Thus, H1 is supported.

**Table 3:** Mediation Effect Test of TAW.

Variables	Outcome variable: TAW		Outcome variable: work engagement		Outcome variable: work engagement	
	β	CI	β	CI	β	CI
Gender	−0.043	[−0.279,0.266]	0.218	[0.104,0.539]	0.241	[−0.063,0.545]
Age	−0.034	[−0.322,0.314]	0.010	[−0.446,0.445]	0.018	[−0.402,0.439]
Education	−0.052	[−0.235,0.101]	0.014	[0.181,0.209]	0.042	[−0.143,0.228]
organizational tenure	0.092	[−0176,0.325]	0.089	[−0.198,0.377]	0.039	[−0.235,0.314]
Position	0.025	[−0.263,0.303]	−0.017	[−0.353,0.319]	−0.031	[−0.347,0.286]
Conscientiousness	0.303	[0.076,0.471]	0.591	[0.302,0.880]	0.326	[0.120,0.731]
TAW					0.445	[0.091,0.999]
R^2^	0.254		0.333		0.423	
*F*-value	2.214^**^		3.244^*^		3.975^**^	

*N* = 322. *p<0.05. **p<0.01.

Second, we tested the mediating effect of workload. As indicated in Table 4, conscientiousness had a significantly positive impact on workload (*β* = 0.388, 95% CI [0.085, 0.860]), and the negative indirect effect of conscientiousness on work engagement via workload was significant (*β* = −0.336, 95% CI [−0.504, −0.168]). Thus, H2 is supported. Furthermore, when TAW and workload were simultaneously included as mediating variables, the results (Table 4) show that the indirect effect of conscientiousness on work engagement via both TAW (*β* = 0.493, 95% CI [0.128, 0.557]) and workload (*β* = −0.219, 95% CI [−0.399,-0.109]) was significant. Therefore, H1 and H2 were further validated.

**Table 4:** The mediation effect of workload and double mediation effect test.

Variables	Outcome variable: workload		Outcome variable: work engagement		Outcome variable: work engagement	
	β	95% CI	β	95% CI	β	95% CI
Gender	−0.160	[−0.986,0.066]	0.063	[−0.220,0.346]	0.082	[−0.171,0.335]
Age	−0.052	[−0.280,0.177]	−0.186	[−0.574,0.203]	−0.173	[−0.020,0.174]
Education	0.067	[−0.250,0.386]	0.036	[−0.129,0.202]	0.068	[−0.081,0.217]
organizational tenure	0.032	[−0.139,0.802]	0.201	[−0.050,0.451]	0.151	[−0.074,0.176]
Position	0.198	[−0.051,0.047]	0.150	[−0.353,0.319]	0.142	[−0.122,0.107]
Conscientiousness	0.388	[0.085,0.860]	0.722	[−0.146,0.447]	0.547	[0.297,0.797]
Workload			−0.336	[−0.504,-0.168]	−0.219	[−0.399,-0.109]
TAW					0.493	[0.128,0.557]
R^2^	0.211		0.534		0.639	
F-value	1.736^*^		6.215^***^		1.736^***^	

*N* = 322. *p<0.05. **p<0.01. ***p<0.001.

#### 4.3.2 Moderation effects and moderated mediation test

**Figure 2:**
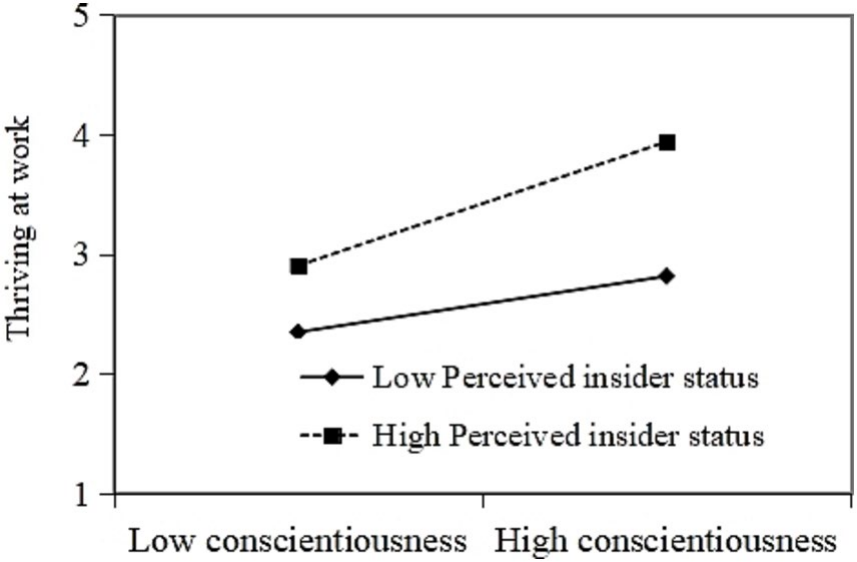
Moderating role of PIS in the relationship between conscientiousness and TAW.

**Figure 3:**
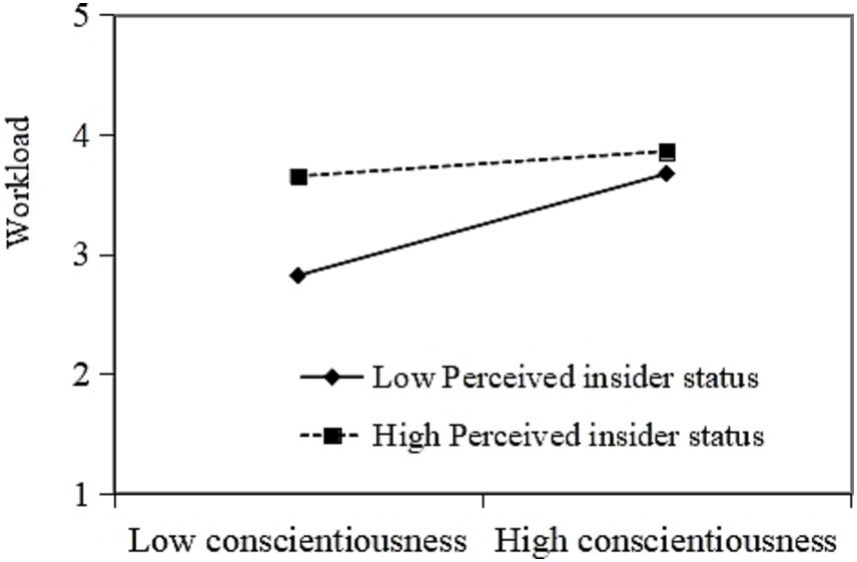
Moderating role of PIS in the relationship between conscientiousness and workload.

Further, we use Model 7 in the Process to test the moderation and moderated mediation effects. The interactive effects of conscientiousness and PIS on TAW were positive and significant (*β* = 0.142, 95% CI (0.004, 0.301)). Thus, H3 is supported. The plot in Figure 2 shows that when PIS is higher, conscientiousness is more positively associated with TAW. In addition, the interactive effects of conscientiousness and PIS on workload were negative and significant (*β* = −0.161, 95% CI (−0.245, −0.062)). Thus, H4 is supported. The plot in Figure 3 indicates that when PIS was lower, conscientiousness was more positively associated with workload.

The moderated mediation effects for H5 and H6 were tested, and the results are presented in Table 5. The differences between the indirect effects of conscientiousness on work engagement via TAW (moderated mediation index = 0.101, 95%CI [0.063, 0.621]) and workload (moderated mediation index = 0.352, 95%CI [0.078, 0.415]) at higher and lower PIS were both significant. Therefore, H5 and H6 are supported.

**Table 5:** Results of moderated mediation tests.

Path	Estimate(SE)	95% CI
Conscientiousness→ TAW→ Work engagement		
High PIS(M + SD)	0.318(0.216)	[0.129,0.879]
Low PIS (M-SD)	0.017(0.161)	[−0.130,0.156]
Estimate difference	0.101(0.115)	[0.063,0.621]
Conscientiousness→ workload→ Work engagement		
High PIS(M + SD)	−0.024	[−0.323,0.093]
Low PIS (M-SD)	−0.376	[−0.578,-0.184]
Estimate difference	0.352	[0.078, 0.415]

## 5 Conclusion and general discussion

### 5.1 Theoretical contributions

First, this study enriches the existing research findings on conscientiousness and employee work engagement. The conscientiousness and work engagement of medical staff in China are closely correlated with the quality of medical care and doctor–patient relationships. Scholars have investigated the correlation between conscientiousness and research productivity (40), well-being (4), and other factors. However, limited research has specifically connected conscientiousness to the work engagement of medical staff. This study added variables such as workload and work engagement to the comprehensive model of conscientiousness, expanding the research on outcome variables, and provided a new theoretical perspective on how conscientiousness as a personality trait drives the work engagement of medical staff.

Second, this study revealed the double-edged sword effect of conscientiousness on employee work engagement and its impact process. Previous studies focused predominantly on the positive impact of conscientiousness on individual work states (4, 6). Recent research in personality theory has introduced the “too-much-of-a-good-thing effect” in the relationship between conscientiousness and ideal outcomes, challenging the “more is better” view that has been dominating research on this trait for a long time (41). However, little is known regarding how conscientiousness leads to negative outcomes (9). We develop an integrative model that provides a balanced and dialectical understanding of the impact of conscientiousness. It simultaneously promotes our understanding of the consequences of conscientiousness from the “bright” and “dark” sides. This study fills a research gap regarding the effectiveness of the impact of conscientiousness and provides a broader perspective on the systematic comprehension of the role of conscientiousness in academia. These findings also expanded the application of COR theory by sustaining the view that not all behaviors deemed “good” can realize resource enhancement (42).

Third, we expand the boundary condition of the conscientiousness–work engagement linkage. China emphasizes collectivism and PIS has research significance in the Chinese cultural context (43). This study introduced PIS as a moderating variable, providing new boundary conditions for exploring the relationship between conscientiousness and medical staff work states. It confirms the impact of PIS on the relationships between conscientiousness, TAW/workload, and work engagement, enriching research that uses PIS as a moderating variable. This provides a new perspective for improving TAW of medical staff and providing interventions in their workloads. However, the integration of PIS with personality trait factors provides important insights for future research on its effectiveness.

### 5.2 Practical implications

First, public hospitals should stimulate a sense of conscientiousness among medical staff. In organizational environments, conscientiousness significantly affects employees’ work attitudes, behaviors, and performance. However, this impact may be negative. Therefore, organizations need to provide relevant training to employees to strengthen their passion for their profession and work. Simultaneously, it is important to remind employees to be mindful of resource depletion, while focusing on their work and to manage their sense of conscientiousness more dynamically.

Second, public hospitals should create a working environment conducive to improving the TAW and reducing the experience of workload for medical staff. On the one hand, creating a working atmosphere characterized by respect and trust from superiors and colleagues, expanding avenues for accessing work-related information, optimizing information dissemination methods, establishing an environment conducive to sharing information and resources, and enabling medical staff to achieve their ideal work state. Public hospitals should employ measures to prevent and intervene in the high workload of medical staff and reduce the perceived burden caused by emotional, physical, and mental resource depletion, thereby lowering the burden efficiency caused by a sense of conscientiousness. Specific measures may include (1) collaborating with the HRM departments of public hospitals to establish relevant support mechanisms, such as stipulating that medical staff must take paid leave each year and generally do not allow explicit or implicit overtime; (2) providing employees with health promotion programs and regularly conducting group/individual physical and mental intervention measures; and (3) establishing an adequate pool of specialized and flexible human resources to address high-intensity medical service work at any time.

Finally, public hospitals should consider PIS as a self-concept factor. Medical staff with higher levels of PIS are better equipped to experience work vitality and prosperity guided by conscientiousness. They can handle job tasks calmly, reduce the sense of being overwhelmed, and increase their levels of work engagement. Therefore, public hospitals can improve the PIS of medical staff by granting them more autonomy, enhancing their professional skills in performing job tasks, assisting them in overcoming work difficulties, creating more career opportunities, and ultimately boosting their PIS.

### 5.3 Limitations and future research directions

This study reached a relatively comprehensive conclusion through empirical analysis; however, it still has limitations that require further refinement in future research. First, the use of homogeneous data may have introduced bias. Despite the tests showing that the CMV is not severe, future research should use multisource and longitudinal data to minimize such biases. Second, contextual factors such as organizational context, leadership styles, and job characteristics may also moderate employees’ work attitudes and behaviors (44). Future studies could use contextual factors such as organizational support and environmental uncertainty (45) as moderating variables to further explore the boundary conditions that influence conscientiousness. Third, due to the limitations of the study conditions, the sample was drawn from only four public hospitals in China. Van et al. (46) noted that individuals’ cultural contexts influence their informational and cognitive processes, which drives the construction of specific cognitive interpretative models. Therefore, future research should consider collecting data from other countries, while selecting other specific industries (such as education and legal practice) to enhance sample diversity and improve the generalizability of the research results.

## 6 Conclusion

Based on the COR and social exchange theory, we investigated the double-edged sword effect of conscientiousness on work engagement among medical staff in public hospitals. The results indicate that (1) conscientiousness has a positive indirect effect on work engagement via TAW, and the indirect effect is stronger when PIS is higher than when lower; furthermore, (2) conscientiousness has a negative indirect effect on work engagement via workload, and the indirect effect is stronger when PIS is lower than when it is higher. Given that the literature on the double-edged sword effect of conscientiousness is still in its infancy, we hope that our study is a step toward gaining a more complete understanding of conscientiousness and their effects on employee work states.

## Data Availability

The raw data supporting the conclusions of this article will be made available by the authors, without undue reservation.
